# Failure to control hypercholesterolaemia in the Irish adult population: cross-sectional analysis of the baseline wave of The Irish Longitudinal Study on Ageing (TILDA)

**DOI:** 10.1007/s11845-017-1590-3

**Published:** 2017-03-10

**Authors:** C. Murphy, E. Shelley, A. M. O’Halloran, T. Fahey, R. A. Kenny

**Affiliations:** 1 School of Nursing and Human Sciences, Dublin City University, Dublin, Ireland; 2 Department of Public Health, Health Service Executive, Dublin, Ireland; 3 The Irish Longitudinal Study on Ageing (TILDA), Department of Medical Gerontology, Trinity College, Dublin, Ireland; 4 HRB Centre for Primary Care Research, Department of General Practice, Royal College of Surgeons in Ireland Medical School, Dublin, Ireland

**Keywords:** Hypercholesterolaemia, Dyslipidaemia, Older adults, Cardiovascular disease, CVD risk

## Abstract

**Background:**

Hypercholesterolaemia is an important modifiable risk factor for cardiovascular disease (CVD) which requires monitoring and management at a population level.

**Aims:**

This study aims to describe the distribution of serum cholesterol in a community living population of older adults in Ireland and to examine the awareness, treatment and control of hypercholesterolaemia according to CVD risk status.

**Method:**

This is a cross-sectional study in a nationally representative sample of adults aged 50–79 years (*n* = 5287). Hypercholesterolaemia was defined as low-density lipoprotein cholesterol (LDL-C) in excess of the recommended CVD risk category target and/or on lipid-lowering medication.

**Results:**

This study reports a mean total cholesterol (TC) of 5.1 mmol/L (95% CI 5.0–5.1 mmol/L) and a mean LDL-C of 2.9 mmol/L (95% CI 2.8–2.9 mmol/L) in those aged 50–79 years. In a subgroup aged 50–64 years, 73% (95% CI 71.5–74.5%) were hypercholesterolaemic. LDL-C was controlled to the guideline target in 57% of those with CVD and 49% of those with diabetes. Lack of awareness of hypercholesterolaemia was high across the remainder of the population.

**Conclusion:**

Despite a substantial reduction in population mean TC from a high of 6.0 mmol/L in the 1980s to 5.1 mmol/L, this study reports a failure to control hypercholesterolaemia to recommended risk-stratified targets in the Irish adult population. Recommendations for policy include continued monitoring of those at highest risk and CVD risk assessment in those perceived to be at low risk in order to inform shared decision making in relation to lifestyle modification and medication management.

## Introduction

Although cardiovascular disease is the leading cause of mortality globally [1] and the leading cause of mortality in Ireland accounting for almost a third of all deaths [2], nonetheless, between 1985 and 2006, coronary heart disease mortality decreased by 68% in men and 69% in women [3]. Almost half of the reduction was attributed to improvements in risk factor levels; 24% was attributed to a reduction in population cholesterol levels [3].

Raised serum cholesterol is an important modifiable risk factor for the development of cardiovascular disease. A reduction in total cholesterol of 1 mmol/L is associated with a 33% reduction in ischaemic heart disease mortality in those aged 50–69 years and a reduction of 17% in those aged 70–89 years [4]. At a population level, total cholesterol (TC), low-density lipoprotein cholesterol (LDL-C) and high-density lipoprotein cholesterol (HDL-C) are assessed to characterise abnormal cholesterol levels [5, 6]. Globally mean TC declined by 0.1 mmol/L between 1980 and 2008 with higher income regions including western Europe declining by 0.2 mmol/L for both sexes [7]. Despite an overall downward trend, a reversal of this trend at population level has recently been observed in Sweden between 2008 and 2010 [8].

Reliable population based data are required in order to monitor the management of hypercholesterolaemia, provide evidence for policy and evaluate cardiovascular disease programmes at a national level. The Irish Longitudinal Study on Ageing (TILDA) provides the opportunity to bridge this information gap.

Clinical guidelines on cholesterol reduction are based on overall CVD risk and not on the absolute level of cholesterol with targets identified according to absolute risk status. The aim of this study is to document the distribution of serum cholesterol in a community living population of older adults aged 50–79 years in Ireland and apply clinical guideline targets [9] to examine the awareness, treatment and control of hypercholesterolaemia in a subgroup aged 50–64 years according to their absolute risk of CVD.

## Methods

### Sample design

The Irish Longitudinal Study on Ageing (TILDA) is an ongoing prospective cohort study representative of community living adults in Ireland aged 50 years and older. The sample was recruited based on a national directory of residential addresses using the RANSAM system [10]. Participants were invited to take part in a home-based interview followed by a health assessment conducted by a research nurse in a designated centre or at home. The study uses cross-sectional data from the baseline wave (October 2009 to July 2011). Analyses are based on those aged 50–79 years at baseline who participated in the health assessment and provided a blood sample (*n* = 5287).

### European Society of Cardiology guidelines and CVD risk

The European Society of Cardiology guidelines current during the data collection period recommended TC and LDL-C targets based on absolute CVD risk [9]. The Systematic COronary Risk Estimation (SCORE) based on a European cohort, estimates the 10-year risk of first fatal atherosclerotic event in asymptomatic individuals up to the age of 64 years based on five risk factors: age, sex, systolic blood pressure, total cholesterol and smoking status [11]. Ireland was classified as a “low risk” country based on CVD and diabetes mortality in 2008, therefore the corresponding risk equations were applied to the TILDA data. Individuals in the sub group (50–64 years) already known to be at high risk for CVD i.e. those with known CVD or diabetes were not classified using SCORE. The remaining participants were classified into one of four CVD risk categories; Very high risk (SCORE ≥10%), High risk (SCORE ≥5 and <10%), moderate risk (SCORE ≥1 and <5%) and low risk (SCORE <1%).

### Lipid measurements

Non fasting venous blood was drawn during the health assessment. The TILDA protocol for blood sample collection, processing and storage has been described previously [12]. Briefly, 5 mL of fresh whole blood was collected in Lithium heparin coated tubes and transported to a central processing laboratory in temperature-controlled shipping boxes which maintained the samples at 2–8 °C for up to 48 h. Plasma samples were then isolated in 1 ml aliquots and placed at 2–8 °C for up to 72 h prior to lipid profile analysis. Lipid concentrations were analysed using Siemans Dimension Xpand chemistry analyser.

Total cholesterol was measured using a single reagent, endpoint reaction method that is specific for cholesterol. The assay is optimised against the Centres for Disease Control and Prevention (CDC) and the National Institute of Standards and Technology (NIST) reference methods. The method is standardised against the Abell/Kendall method and isotope dilution/mass spectrometry. The assay met the 1992 National Institutes of Health goal for precision and bias. LDL-C was calculated using the Friedewald formula. LDL-C is underestimated at higher triglyceride concentrations, therefore LDL-C was only calculated on participants with triglyceride values <4.0 mmol/L [13]. A direct determination of high-density lipoprotein cholesterol (HDL-C) was performed using PEG-modified enzymes and dextran sulphate. The Roche HDL-C assay meets the 1998 National Institutes of Health/National Cholesterol Program (NCEP) for acceptable performance. The results of this method correlate with those obtained by precipitation-based methods and also by ultracentrifugation. The method has been standardised against the designated CDC reference method. The standardisation meets the requirements of the “HDL-Cholesterol Method Evaluation Protocol for Manufacturers” of the US National Reference System for Cholesterol, CRMLN (Cholesterol Reference Method Laboratory Network), 1994.

In the subgroup aged 50–64, hypercholesterolaemia was defined as a LDL-C in excess of the risk category target as outlined in the guidelines (Table 3) and/or on lipid-lowering medication. Control of hypercholesterolaemia was defined as LDL-C below the guideline target in conjunction with the use of lipid-lowering medication.

### Measurements

Demographic and social characteristics considered in this analysis include age, sex and highest educational attainment (primary, secondary and tertiary). Lipid-lowering medication use was recorded during the home interview. Medication was classified according to the WHO anatomical therapeutic chemical classification system (ATC), all medications coded C10 were included. Good agreement has been demonstrated in this cohort between lipid-lowering medication recorded at interview and pharmacy dispensing records (*k* = 0.73, 95% CI 0.69–0.77) [14].

Cardiovascular disease (angina, myocardial infarction, heart failure, stroke, transient ischaemic attack, stent/vascular surgery), diabetes and high cholesterol were self-reported based on ever having a doctor’s diagnosis of these conditions. Respondents were also asked if they had ever had a blood test for cholesterol.

Physical activity was self-assessed using the International Physical Activity Questionnaire (IPAQ) short form and categorised into low, moderate or high levels of physical activity [15]. Blood pressure was measured twice during the health assessment and the mean systolic and diastolic blood pressure recorded. Smoking status was self-reported during the home interview. Three mutually exclusive medical insurance categories were used, these include those with (a) a medical card which provides free access to primary care and subsidised medication (b) private health insurance only and (c) no health insurance.

### Statistical analysis

Descriptive statistics are presented at percentages, means and standard deviations. Statistical significance was calculated using *t* tests for continuous variables and Chi-squared tests for categorical variables and set at *p* < 0.05. Statistical weights were applied to the sample to adjust for selection bias and non-response to the health assessment component of the survey. Individual weights were calibrated against the age, sex and educational profile of the Irish population sourced from the Quarterly National Household Survey 2010 compiled by the Irish Central Statistics Office. The subgroup analysis is unweighted. Statistical analysis was performed using the TILDA wave 1 data file v1-7-8 and statistical software Stata/MP V.12.1

## Results

The household response rate to the baseline TILDA survey was 62%. Of the 8175 adults who participated in the TILDA study, 7537 were aged between 50 and 79 years. Of these, 70.1% (*n* = 5287) took part in the health assessment, had complete risk factor data available and were included in the analysis. The included participants were younger; more highly educated and displayed more favourable cardiovascular risk characteristics when compared to those not included in the analysis e.g. smoking (Table 1).

**Table 1:** Characteristics of TILDA participants (TILDA wave 1, 50–79 years)

	All eligible participants (*n* = 7537)	Included in the analysis (*n* = 5287)	Excluded from the analysis (*n* = 2250)	*p* value
Age mean (SD)	62.1 (8.1)	61.7 (7.9)	63.2 (8.5)	<0.001
Male sex (%)	46.0	46.5	45.0	0.236
Education				
Primary	28.5	24.1	38.8	
Secondary	40.9	41.8	38.6	
Tertiary	30.5	33.9	22.4	<0.001
Smoker	19.0	16.1	25.7	<0.001
Physical activity				
Low	30.1	28.6	33.8	
Moderate	34.7	35.3	33.3	
High	35.0	36.0	32.7	<0.001
Health insurance				
No cover	11.1	11.0	11.5	
Private health insurance	43.2	48.5	30.7	
Medical card	45.6	40.4	57.7	<0.001
Known CVD	10.6	10.0	12.2	0.004
Known diabetes	7.4	6.9	8.8	0.003
Lipid-lowering medication	32.6	33.9	29.7	0.001

Included in the analysis: participants were included in the analysis if they took part in the home interview and the health assessment and provided full risk factor data including providing a blood sample

Statistical significance was calculated using *t* test for continuous variables and Chi-square test for categorical variables

*CVD* cardiovascular disease, *TILDA* The Irish Longitudinal Study on Ageing

### Total cholesterol, LDL cholesterol and HDL cholesterol

**Fig. 1:**
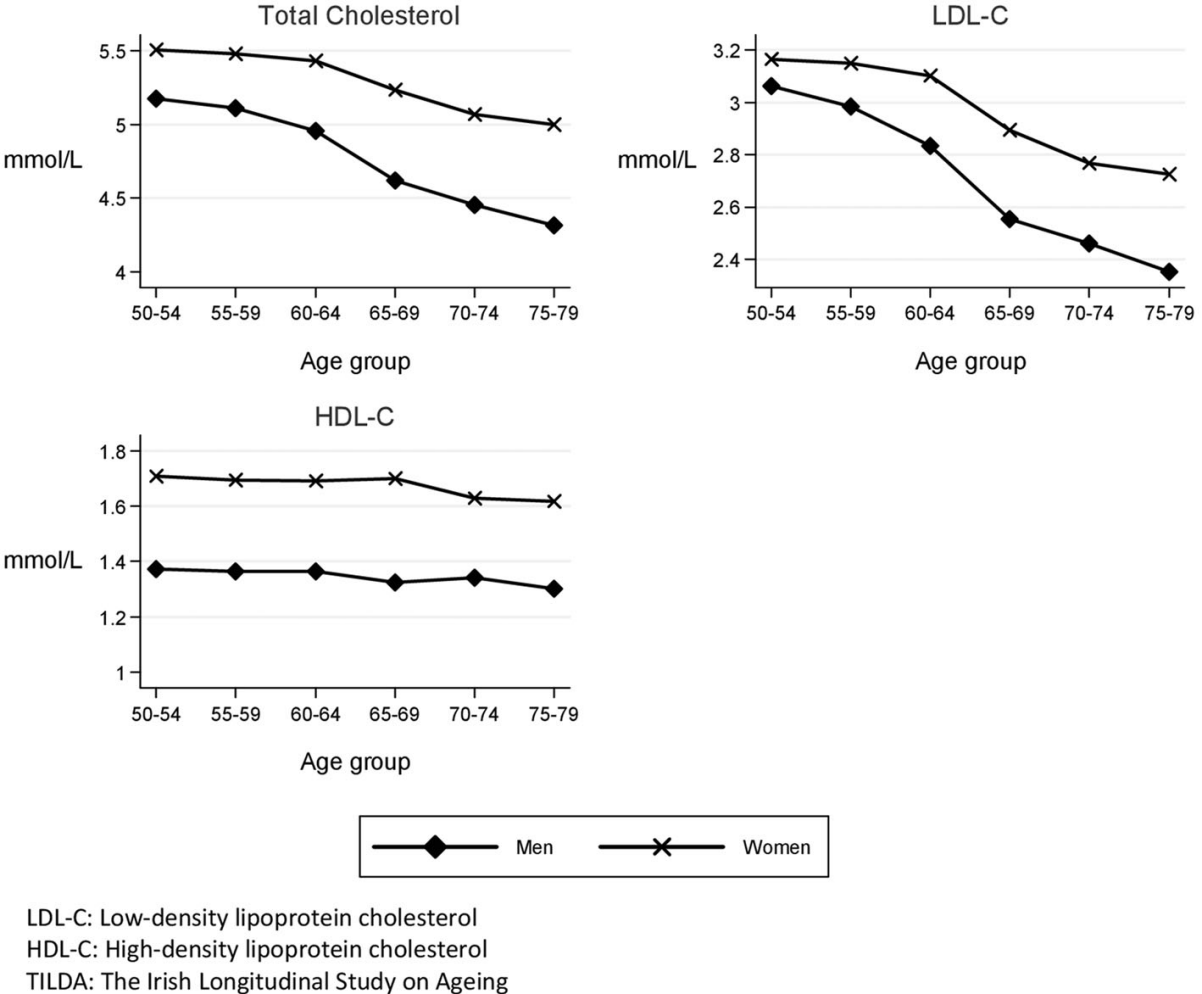
Weighted mean total cholesterol, mean LDL cholesterol and mean HDL cholesterol according to sex and age group (TILDA wave 1, 50–79 years, *n* = 5287)

The weighted mean TC, LDL-C and HDL-C in the population aged 50–79 years are detailed in Table 2. Figure 1 graphically depicts the weighted mean TC, LDL-C and HDL-C according to sex and 5 year age group and shows a decline with advancing years more marked in men than in women for TC and LDL-C, whereas HDL-C remained unchanged with age.

**Table 2:** Weighted total cholesterol, low-density lipoprotein cholesterol and high-density lipoprotein cholesterol according to sex and lipid-lowering treatment status (TILDA wave 1, 50–79 years)

	On LLM	No LLM	All	*n*
	Mean (95% CI)	Mean (95% CI)	Mean (95% CI)	
Total cholesterol, mmol/L				
Male	4.3 (4.2–4.4)	5.1 (5.1–5.2)	4.8 (4.8–4.9)	2459
Female	4.8 (4.8–4.9)	5.5 (5.5–5.6)	5.3 (5.3–5.4)	2828
Total	4.5 (4.5–4.6)	5.3 (5.3–5.4)	5.1 (5.0–5.1)	5287
LDL cholesterol, mmol/L				
Male	2.2 (2.1–2.3)	3.0 (3.0–3.1)	2.7 (2.7–2.8)	2316
Female	2.5 (2.5–2.6)	3.2 (3.1–3.2)	3.0 (2.9–3.0)	2753
Total	2.4 (2.3–2.4)	3.1 (3.1–3.2)	2.9 (2.8–2.9)	5069
HDL cholesterol, mmol/L				
Male	1.3 (1.2–1.3)	1.3 (1.3–1.4)	1.3 (1.3–1.4)	2459
Female	1.6 (1.5–1.6)	1.7 (1.6–1.7)	1.6 (1.6–1.7)	2828
Total	1.4 (1.4–1.4)	1.5 (1.5–1.5)	1.5 (1.5–1.5)	5287

*LLM* lipid-lowering medication, *LDL cholesterol* low-density lipoprotein cholesterol, *HDL cholesterol* high-density lipoprotein cholesterol, *CI* confidence interval, *TILDA* The Irish Longitudinal Study on Ageing

**Fig. 2:**
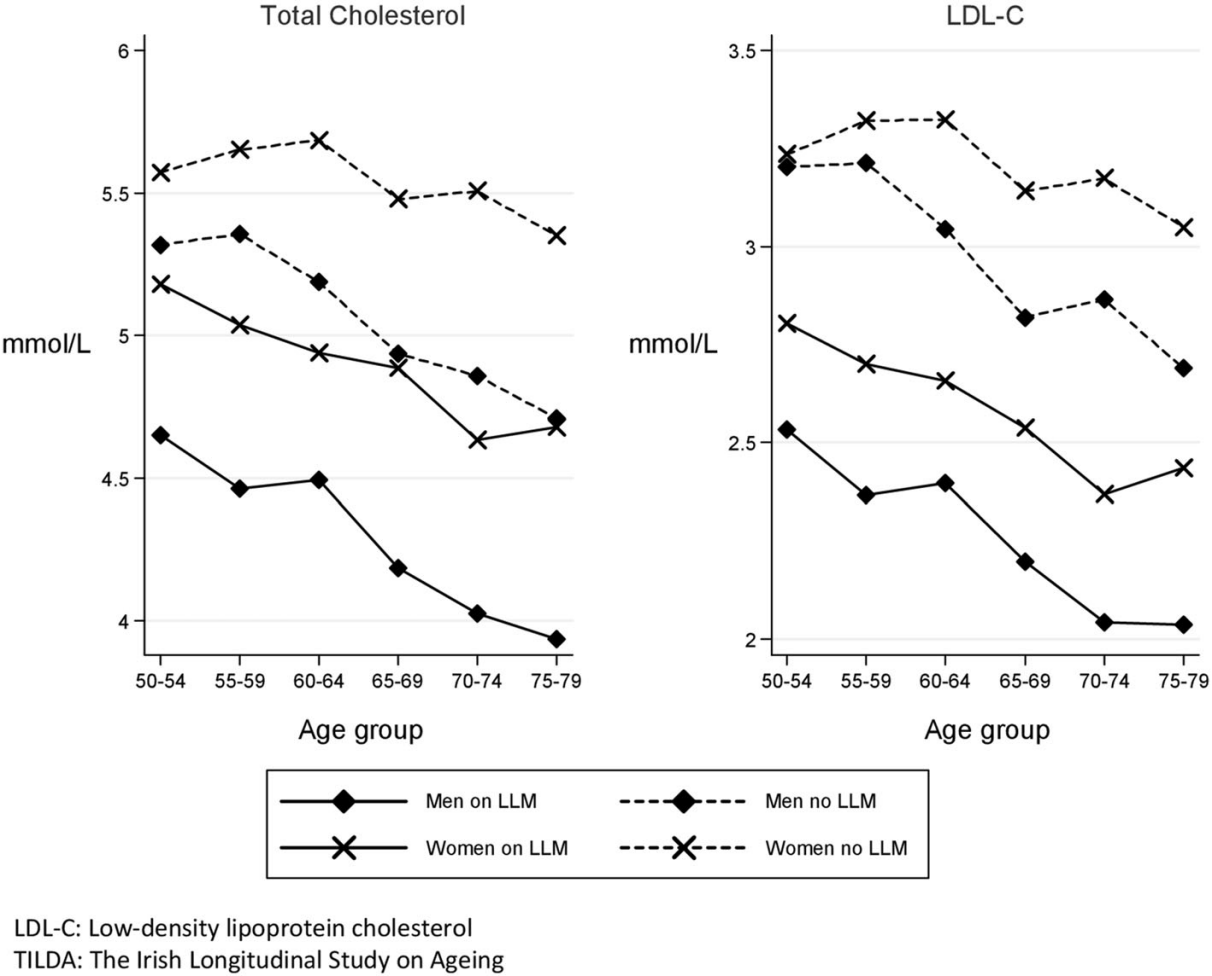
Weighted mean total cholesterol, mean LDL cholesterol according to sex and age group and lipid-lowering medication status (TILDA wave 1, 50–79 years, *n* = 5287)

LLM was used by 33.9% (95% CI 32.3–35.4%) of the sample. This proportion varied significantly across medical insurance categories. A higher proportion of those with a medical card (42.1%, 95% CI 39.7–44.5%) were on LLM compared to 28.4% (95% CI 26.3–30.4%) of those with private insurance only and 22.5% (95% CI 18.8–26.1%) of those with no medical insurance. This difference persisted after adjustment for age, sex and educational status. In those not taking LLM, the weighted mean TC was 5.3 mmol/L (95% CI 5.3–5.4), 5.1 mmol/L (95% CI 5.1–5.2) in men and 5.5 mmol/L (95% CI 5.5–5.6) in women (Table 2). In this group (not on LLM) the weighted mean TC and LDL-C peaked in men aged 55–59 years and in women aged 60–64 years (Fig. 2).

A high proportion of participants 88.9% (95% CI 87.8–89.9%) reported ever having a blood test for cholesterol. This proportion varied significantly with age from 82.6% (95% CI 80.1–85.1%) in the 50-54 year age group to 94.4% (95% CI 91.8–96.9%) in the 75–79 year age group. Equal proportions of men and women self-reported a blood test for cholesterol. In total, 41.1% (95% CI 39.6–42.7%) reported ever having a doctor’s diagnosis of high cholesterol.

Almost half of the population aged 50–79 years (44.8%, 95% CI 43.3–46.3%) had a TC <5 mmol/L and just over half (51.3%, 95% CI 49.8–52.8%) had an LDL-C <3 mmol/L. Only 22.3% (95% CI 21.1–23.5%) had a TC <5 mmol/L and were not on LLM, and 26.6% (95% CI 25.3–27.9%) had an LDL-C <3 mmol/L and were not on LLM.

### Hypercholesterolaemia according to CVD risk status

A subgroup analysis was conducted on those aged 50–64 years, in whom absolute risk based on SCORE or morbidity status could reliably be calculated (*n* = 3227). This population were categorised into six groups: known CVD 4.8% (*n* = 155), know diabetes 4.2% (*n* = 135), very high SCORE risk 0.6% (*n* = 20), high SCORE risk 3.4% (*n* = 109), moderate SCORE risk 49.2% (*n* = 1587) and low SCORE risk 37.8% (*n* = 1221). The very high SCORE risk group and the high SCORE risk group were combined into a single group (*n* = 129) due to the small number in the very high risk group and the identical LDL-C targets for these two groups in the clinical guidelines.

Targets for LDL-C according to CVD risk are identified in Table 3, column one [9]. Hypercholesterolaemia prevalence (LDL-C above CVD risk category target and/or on LLM) was 73% (95% CI 71.5–74.5%) (*n* = 2357) overall with the prevalence ranging from 66.6% (95% CI 64.0–69.3%) in the low risk group to 90.9% (95% CI 86.4–95.5%) in those with known CVD.

**Table 3:** Hypercholesterolaemia prevalence according to CVD risk status in adults aged 50–64 years (TILDA wave 1)

	Target LDL-C^a^	LDL-C	Hypercholesterolaemia	Total
	mmol/L	Mean (SD)	% (95% CI)	*n*
Known CVD	<2.5	2.3 (1.0)	90.9 (86.4–95.5)	155
Known diabetes	<2.5	2.4 (1.0)	86.6 (80.8–92.4)	135
Very high risk and high risk	<2.5	3.4 (1.0)	89.9 (84.6–95.1)	129
Moderate risk	<3.0	3.1 (0.9)	73.6 (71.4–75.8)	1587
Low risk	<3.0	3.0 (0.8)	66.6 (64.0–69.3)	1221
Total	–	3.0 (0.9)	73.0 (71.5–74.5)	3227

Very high and high risk = SCORE ≥5%, Moderate risk = SCORE ≥1 and <5%, Low risk = SCORE <1%

^a^2007 Guidelines [9]

*CVD* cardiovascular disease, *LDL-C* low-density lipoprotein cholesterol, *SD* standard deviation, *CI* confidence interval, *TILDA* The Irish Longitudinal Study on Ageing, *Hypercholesterolaemia* LDL-C above target for risk group and/or on lipid-lowering medication

**Fig. 3:**
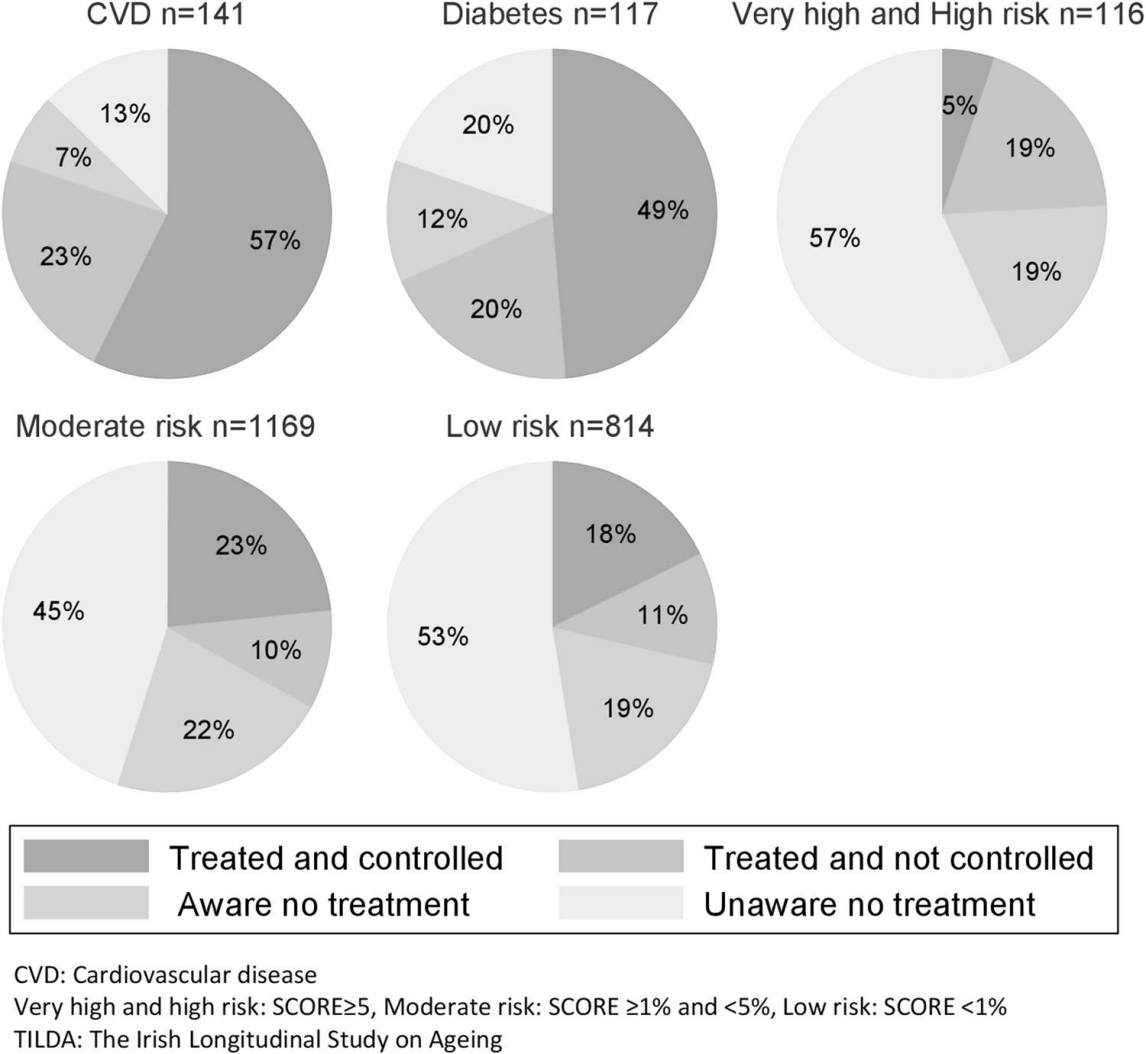
Awareness treatment and control of hypercholesterolaemia according to cardiovascular disease risk status (TILDA wave 1, 50–64 years)

Examination of those with hypercholesterolaemia (*n* = 2357) revealed a varied picture in relation to awareness, treatment and control of LDL-C depending on the absolute level of CVD risk of individual participants (Fig. 3). Over half of those with known CVD and close to half of those with known diabetes (49%) were treated and controlled to the recommended LDL-C target level. This contrasts with the small proportion (5%) treated and controlled to target in the very high/high SCORE risk group. Lack of awareness of hypercholesterolaemia was lowest in those with existing disease (CVD and Diabetes) and high across all SCORE risk groups.

## Discussion

The study reports a weighted mean TC of 5.1, 4.8 mmol/L in men and 5.3 mmol/L in women and a weighted mean LDL-C of 2.9, 2.7 mmol/L in men and 3.0 mmol/L in women in a nationally representative sample of adults aged 50–79 years in the Irish population during the period 2009–2011. One-third of adults in the age group were on lipid-lowering medication. In a subgroup aged 50–64 years, the prevalence of hypercholesterolaemia was 73%. Despite European and National policy recommendations and guidelines regarding the risks associated with high cholesterol [9, 16], control of LDL-C in those at highest risk for a CVD event is lower than expected with 57% of those with known CVD and 49% of diabetics achieving guideline targets. Lack of awareness of hypercholesterolaemia was high across all primary prevention risk groups.

These results compare with findings from a nationally representative cohort of non-institutionalised adults aged 20 years and older in the National Health and Nutrition Examination Surveys (NHANES) in the United States which found an overall TC of 196 mg/dL (equivalent to 5.0 mmol/L) and LDL-C of 116 mg/dL (equivalent to 3.0 mmol/L) during the time period 2007–2010 [17]. The Health Survey for England 2011 conducted in a community living sample of adults aged 16 years and older found a mean TC of 5.1 mmol/L in men and 5.2 mmol/L in women [18]. Differences in the age profile of participants in other studies creates difficulty in making direct comparisons, however, certain patterns in the data are similar to those found elsewhere. Women had higher cholesterol levels than men at all ages beyond 50 years in our study. This is similar to other studies of younger cohorts which found that up to the age of 50 years men had higher cholesterol levels than women, however, once over the age of 50 years women have higher cholesterol levels [19]. The finding that cholesterol levels peak higher and later in women compared to men is consistent with findings from representative surveys of the French population in 2006/2007 [20] and the Health Survey for England 2011 [19]. This unique rise in cholesterol in peri-menopausal women is also documented in the Study of Women’s Health across the Nation (SWAN) in the United States which highlights the importance of monitoring lipids in older women [21].

Previously in Ireland, the Kilkenny health project conducted TC examinations at baseline on an intervention and reference group (aged 35–64 years) in 1985–1986. Mean total cholesterol in men was 6.0 mmol/L in the intervention and in the reference group and 6.0 mmol/L in women in the intervention group and 5.9 mmol/L in the reference group [22]. While our study examines TC in an older cohort (50–79 years) it identifies a substantial decline in TC of approximately 0.9 mmol/L in the population over a 25 year period and may reflect a survivorship effect. This decline exceeds the 0.2 mmol/L decline in TC per decade in the European region as outlined by Farzadfar et al. [7] and is similar to results from the northern Sweden MONICA study between 1994 and 2014, where cholesterol levels decreased by 0.7 mmol/L in the 20 year period [23]. Previous research on a sample of 1207 adults aged 45 years and older taken from a nationally representative sample of adults in Ireland found that 18% of the population had a TC <5 mmol/L and were not on lipid-lowering medication [24]. Our study found an increase in this proportion to 22%. This may be partly due to our larger sample size, slightly older age profile or an improvement in dietary and lifestyle factors in the population during the intervening time period.

The use of cholesterol-lowering medication in this cohort (33%) was slightly higher than that reported by the National Health and Nutrition Examination Survey (NHANES) during 2011–2012 when 28% of adults aged 40 years and older were reported using prescription cholesterol-lowering medication in the previous 30 days [25].

The findings in the subgroup aged 50–64 years present an opportunity to examine interventions aimed at lowering cholesterol according to an individual’s CVD risk profile. In individuals at high risk of future CVD events i.e. those with existing CVD or diabetes, opportunities are being missed to achieve LDL-C control as recommended in clinical guidelines. These findings corroborate those found in an observational cohort of Irish patients during the same time period which found persistent dyslipidaemia despite statin therapy [26]. Our findings also reveal a low level of treatment and control and lack of awareness in those classified as very high risk (SCORE ≥10%) or high risk (SCORE ≥5 and <10%). Providing risk information has been shown to change high risk patients perception of risk and physician's prescribing habits [27] yet recent research from Ireland found that only a third of general practitioners (GPs) frequently use a CVD risk assessment tool in practice [28]. Our data is limited in that we collected information on current medication use and therefore cannot ascertain prescription of lipid-lowering medication and the extent to which non adherence to treatment is a feature of our data. Individuals at lower absolute CVD risk are required to weigh up the smaller benefits to them individually of taking medication and the adoption of lifestyle modification in consultation with their general practitioner (GP). In one UK study following the widening of the risk threshold for offering statin therapy one in four patients took up the opportunity to discuss starting statins with their GP and only one in ten chose to start a statin [29]. Poor adherence to statins is reported in primary prevention populations with low perceived risk a strong predictor of poor adherence and many displaying a preference for dietary control [30]. Our findings reveal a lack of awareness of hypercholesterolaemia in the primary prevention population including those at low, moderate and high absolute risk. The largest number of future cases of CVD will arise in the moderate risk group due to its relative size. Our findings suggest that opportunities to promote dietary and lifestyle modification at a population level to reduce future CVD are being missed.

The strengths of this study include the large nationally representative sample which allows us to generalise our findings to the older adult population in Ireland. Additional strengths include the use of standardised protocols for the interviews, health assessments and laboratory methods. The TC and LDL-C levels reported may underestimate the cholesterol profile in this population as those included in the analysis had more favourable risk profiles than respondents who did not take part in the health assessment and thus were excluded from the analysis. This selection bias was addressed at the design stage by the inclusion of home and health centre based assessments and at the analysis stage by the utilisation of calibration weights. Information on cholesterol testing and awareness of hypercholesterolaemia was based on self-report which may be subject to misclassification. In this study we used the SCORE risk assessment tool and associated guideline LDL-C target thresholds. However, a large number of CVD risk assessment tools are in use in primary care [27, 31]. While most recommend prediction models based on simple risk factor data for decision making or further investigation and management, there is no consensus on the strategy for screening, recommended target populations, screening tests or treatment thresholds [31]. We acknowledge the uncertainty this creates in using a single risk assessment tool in our analysis.

Our findings show a decrease in TC and LDL-C in line with global trends which are likely driven by improvements in diet and pharmacological interventions which reduce cholesterol. Despite these improved results at a population level, our analysis of the levels of awareness, treatment and control of hypercholesterolaemia within CVD risk categories demonstrates an ongoing need for targeted interventions to reduce LDL-C especially in those at highest risk of CVD in the older Irish population.
